# Facile synthesis of Fe_2_O_3_, Fe_2_O_3_@CuO and WO_3_ nanoparticles: characterization, structure determination and evaluation of their biological activity

**DOI:** 10.1038/s41598-024-55319-8

**Published:** 2024-03-13

**Authors:** Asmaa T. Mohamed, Reda Abdel Hameed, Shahira H. EL-Moslamy, Mohamed Fareid, Mohamad Othman, Samah A. Loutfy, Elbadawy A. Kamoun, Mohamed Elnouby

**Affiliations:** 1 Nanotechnology Research Center (NTRC), The British University in Egypt, El-Shorouk City, Suez Desert Road, P.O. Box 43, Cairo, 11837 Egypt; 2 Basic Science Department, Preparatory Year, University of Ha’il, 1560 Hail, Saudi Arabia; 3 Medical and Diagnostic Research Centre, University of Ha’il, 55473 Ha’il, Saudi Arabia; 4 Bioprocess Development Department, Genetic Engineering and Biotechnology Research Institute (GEBRI), City of Scientific Research and Technological Applications (SRTA-City), New Borg Al-Arab City, 21934 Alexandria Egypt; 5 Virology and Immunology Unit, Cancer Biology Department, National Cancer Institute (NCI), Cairo University, Fom El-Khalig, 11796 Cairo Egypt; 6 Department of Chemistry, College of Science, King Faisal University, 31982 Al-Ahsa, Saudi Arabia; 7 Polymeric Materials Research Department, Advanced Technology and New Materials Research Institute (ATNMRI), City of Scientific Research and Technological Applications, New Borg Al-Arab City, 21934 Alexandria Egypt; 8 Nanotechnology and Composite Materials Department, Advanced Technology and New Materials Research (ATNMRI), City of Scientific Research and Technological Applications (SRTA-City), New Borg Al-Arab City, 21934 Alexandria Egypt

**Keywords:** Fe_2_O_3_, Fe_2_O_3_@CuO, WO_3_, Biological activity evaluations, Functionalized nanomaterials, Antiviral, Anticancer, Medical applications, Biochemistry, Biological techniques, Cancer, Biogeochemistry, Chemistry, Materials science, Physics

## Abstract

Due to their high specific surface area and its characteristic’s functionalized nanomaterials have great potential in medical applications specialty, as an anticancer. Herein, functional nanoparticles (NPs) based on iron oxide Fe_2_O_3_, iron oxide modified with copper oxide Fe_2_O_3_@CuO, and tungsten oxide WO_3_ were facile synthesized for biomedical applications. The obtained nanomaterials have nanocrystal sizes of 35.5 nm for Fe_2_O_3_, 7 nm for Fe_2_O_3_@CuO, and 25.5 nm for WO_3_. In addition to octahedral and square nanoplates for Fe_2_O_3_, and WO_3;_ respectively. Results revealed that Fe_2_O_3_, Fe_2_O_3_@CuO, and WO_3_ NPs showed remarked anticancer effects versus a safe effect on normal cells through cytotoxicity test using MTT-assay. Notably, synthesized NPs *e.g.* our result demonstrated that Fe_2_O_3_@CuO exhibited the lowest IC_50_ value on the *MCF-7* cancer cell line at about 8.876 µg/ml, compared to Fe_2_O_3_ was 12.87 µg/ml and WO_3_ was 9.211 µg/ml which indicate that the modification NPs Fe_2_O_3_@CuO gave the highest antiproliferative effect against breast cancer. However, these NPs showed a safe mode toward the *Vero* normal cell line, where IC_50_ were monitored as 40.24 µg/ml for Fe_2_O_3_, 21.13 µg/ml for Fe_2_O_3_@CuO, and 25.41 µg/ml for WO_3_ NPs. For further evidence. The antiviral activity using virucidal and viral adsorption mechanisms gave practiced effect by viral adsorption mechanism and prevented the virus from replicating inside the cells. Fe_2_O_3_@CuO and WO_3_ NPs showed a complete reduction in the viral load synergistic effect of combinations between the tested two materials copper oxide instead of iron oxide alone. Interestingly, the antimicrobial efficiency of Fe_2_O_3_@CuO NPs, Fe_2_O_3_NPs, and WO_3_NPs was evaluated using *E. coli, S. aureus,* and *C. albicans* pathogens. The widest microbial inhibition zone (*ca.* 38.45 mm) was observed with 250 mg/ml of WO_3_ NPs against *E. coli*, whereas using 40 mg/ml of Fe_2_O_3_@CuO NPS could form microbial inhibition zone *ca.* 32.86 mm against *S. aureus*. Nevertheless, *C. albicans* was relatively resistant to all examined NPs. The superior biomedical activities of these nanostructures might be due to their unique features and accepted evaluations.

## Introduction

Novel nanomaterials and advanced nanotechnologies prompt the fast development of new protocols for biomedical applications. Ferric oxide, ferric oxide modified with copper oxide and tungsten oxide attracts great attention, due to their (biomedical application), another remarkable feature of ferric oxide and tungsten oxide nanoparticles is their selective toxicity to cancer cells, which opens the way for a new promising pathway for treatment^1^. Recently, there is an important aspect of magnetic nanoparticles in relation to biomedical applications through their biocompatibility and chemical stability which play a great role in the antiviral, antimicrobial, and anticancer effects^2^. The interaction of MNPs with the biological system has the possibility of the target drug delivery and cancer therapy through escaping from the Reticuloendothelial System (RES) which forms part of the immune system^3^. Increasing the time of circulation makes them more effective and preferable than the traditional ways of cancer therapy as chemotherapy^4,5^. enhances their accumulation at the tumor site rather than the free anticancer drug which causes severe side effects^6^. Recent studies have elucidated that MNPs induce cancer cell ablation^7^ by induction of cell apoptosis with limited toxicity to normal cells^8^, (DNA fragmentation and caspase activation are the main hallmarks of apoptosis) with other mechanisms such as nanoparticle-mediated necrosis and autophagy^9^, which added advantage to the concept of the traditional ways of anticancer drugs^10^. Cancer is a critical disease that scientists are interested in since it has a long history of being one of the major causes of mortality. Cancer is not a single condition; rather, it is a group of disorders characterized by unregulated cell development. Tumors are a severe risk of lethal disease that has no geographic or organ limits; they cause an annual global mortality of more than 12.7 million people. Tumor illnesses are often caused by mutations in genes that regulate growth and are involved in DNA repair, cell division, and death.

Iron oxide (Fe_2_O_3_) magnetic nanoparticles have many roles in biomedical applications; their chemical stability, biocompatibility, and size as nanoscales can be used as targeted therapy, cell leveling, repairing tissue, and hyperthermia^11^ in addition to their anticancer, antibacterial, and antiviral mechanisms^12^. These nanoparticles' exceptional ability to trace and then eliminate cancer cell potentials ensures their uniqueness. Heating systems that rely on Fe3O4 to control the release of medications from their delivery system can also be used^13^. These systems are designed to release their substance at temperatures, which highlights further benefits of using the NPs-dependent delivery method in conjunction with the hyperthermia modality for cancer treatment. clinical diagnosis as magnetic fluid hyperthermia^14^ (MFH) and their magnetic resonance image (MRI)^11^. Furthermore, their ability to interact with biological factors (*e.g*. virus, pathogen^1^, or other biological targets and increase their signal sensitivity^15^ with particular resolution^16^ so, can be visualized by MRI and MFH^16,17^. Therefore, MNPs have attracted continuous attention due to highly functionalized magnetic nanoparticles^18^ which are able not only to target cancer cells via selective interaction between nanoparticles and cancer cells, especially the tumor mass, but also via their utilization for tumor imaging as a diagnostic approach^7^. Iron oxide nanoparticles were found to make induction of reactive oxygen species (ROS), induce depletion of glutathione^19^ further, reduce the matrix metalloproteinase-2 (MMP-2), loss of mitochondrial membrane potential^20,4^, and activation of caspase-3 which is responsible for cell apoptosis^8,21^. Fe_2_O_3_ NPs were later used as a targeted therapy to reach the tumor mass directly through the bloodstream^3,22^ with a compatible medium that can be dispersed in the form of clusters to prevent their aggregation^18,23^. Moreover, in parallel, MNPs were used as antiviral agents not only to prevent viral infection but also in clinical diagnosis for identifying the virus target^17^. This approach is based on targeting the virus through functionalized nanoparticles, hence preventing its pathogenesis by inhibiting or competing for its attachment to the host cells’ receptors^24^.

Iron oxide modified with copper oxide NPs plays a great role in biological applications, due to their biocompatibility with normal cells and affinity to express their effect on pathogenic cells^25^, copper oxide nanoparticles have an effective role in inducing the anticancer effect of iron oxide nanoparticles through the production of reactive oxygen species (ROS)^26^, lipid peroxidation and genotoxic effect by chromosomal damage^27^ which pushes the cells to the apoptotic pathway in order to achieve tumor mass ablation^27,28^. Copper oxide nanoparticles showed a higher apoptotic effect^29^ and antimetastatic potential^30^ which was accomplished by elevating the cellular reactive species^31^, inhibiting of matrix metallopeptidase 9, and enhancing P53 expression which increases the apoptotic pathway^32,33^. Which assists the anticancer activity of iron oxide nanoparticles.

The antimicrobial effect of copper oxide NPs was determined by releasing Cu^2+^ ions leading to ROS production^34^ and interacting directly with bacterial cells through the cell membrane of a biological target thus, exerting its bactericidal effect^34^.

Tungsten oxide (WO_3_) NPs have a characteristic scale and feature role to be used as an anticancer agent through cell membrane damage, denaturation to proteins, and ROS production with ultimate apoptosis and cell death^35,36^. So, WO_3_ NPs have genotoxic and cytotoxic effects through their interference with DNA and protein of DNA synthesis or attacking the 5th phosphate group of DNA. And already expressed their genotoxic effect through oxidative stress by the production of ROS^37^ , thus leading to damage to the lipid and cell membrane^38,39^. Furthermore, WO_3_ NPs have antimicrobial effects^40^ by interacting with the bacterial cell membrane^39^, followed by the destruction of the bacterial cell^41,42^. Additionally, MNPs have great virucidal activity against human Adenovirus type-5 (HAdV-5)^43^. The ability of the MNPs to block the viral cell surface receptor, prevent viral attachment and viral entry to the cells, and preventing its pathogenesis through. Which, it makes irreversible changes inside the viral genome^42^, meanwhile prevent its replication inside the treated cells^44,45^. The magnetic nanoparticles (MNPs) might be functionalized intensively for great benefits in biomedical applications. However, MNPs like Fe_2_O_3,_ Fe_2_O_3_@CuO, and WO_3_ have not been entirely investigated lately regarding their biological activity evaluations in literature. Herein, this work aims to discuss simple synthesis routes, instrumental characterization, and biological activity evaluations of synthesized MNPs cytotoxicity, antimicrobial, and antiviral activities were assessed and discussed in detail. Our findings clarified the following potential mechanisms for this complex's impact on the used cell lines and explained the efficiency of the synergetic effect between iron oxide with copper oxide.

## Materials and methods

### Materials

#### Nanoparticles preparations

Iron metal powder (> 99%), copper nitrate (> 99%), and NaOH (> 99%) were purchased from Belaqmi Fine Chemicals, India, sodium tungstate (Na_2_WO_4_·2H_2_O, > 98%) was obtained from Sisco, India***,*** ion-exchange resin (Rohm& Haas, France)*.*

*Cell culture: Vero* cell line (normal monkey kidney cells), *MCF-7* cell line (breast cancer cells) was obtained from (Vaccination and Sera Collection Organization (VACSERA), Agouza, Giza, Egypt). Dulbecco’s Modified Eagle Medium basal medium (DMEM) was purchased from (Sigma, Munich, Germany). And supplemented with 10% fetal bovine serum (FBS),1% penicillin–streptomycin (100 IU/ml), 1% l-glutamine, and 3% sodium bicarbonate were purchased from (Gibco, Merelbeke, Belgium). Trypsin–EDTA (0.025% trypsin and 0.0025% EDTA) and PBS (phosphate buffer saline) tablets were purchased from (Fisher Scientific, Loughborough, UK). MTT 3-(4,5-dimethylthiazol-2-yl)-2,5-diphenyltetrazolium bromide was purchased from (SERVA Electrophoresis GmbH, Heidelberg, Germany). Antimicrobial activity was tested using a variety of human pathogens, including *Gram*-negative bacteria (*Escherichia coli*), *Gram*-positive bacteria (*Staphylococcus aureus*), and fungal cells (*Candida albicans*). The tested human pathogens were kindly provided from GEBRI, SRTA-City, Alexandria, Egypt.

### *Synthesis of Fe*_*2*_*O*_*3*_*, Fe*_*2*_*O*_*3*_*@CuO and WO*_*3*_* NPs*

**Scheme 1:**
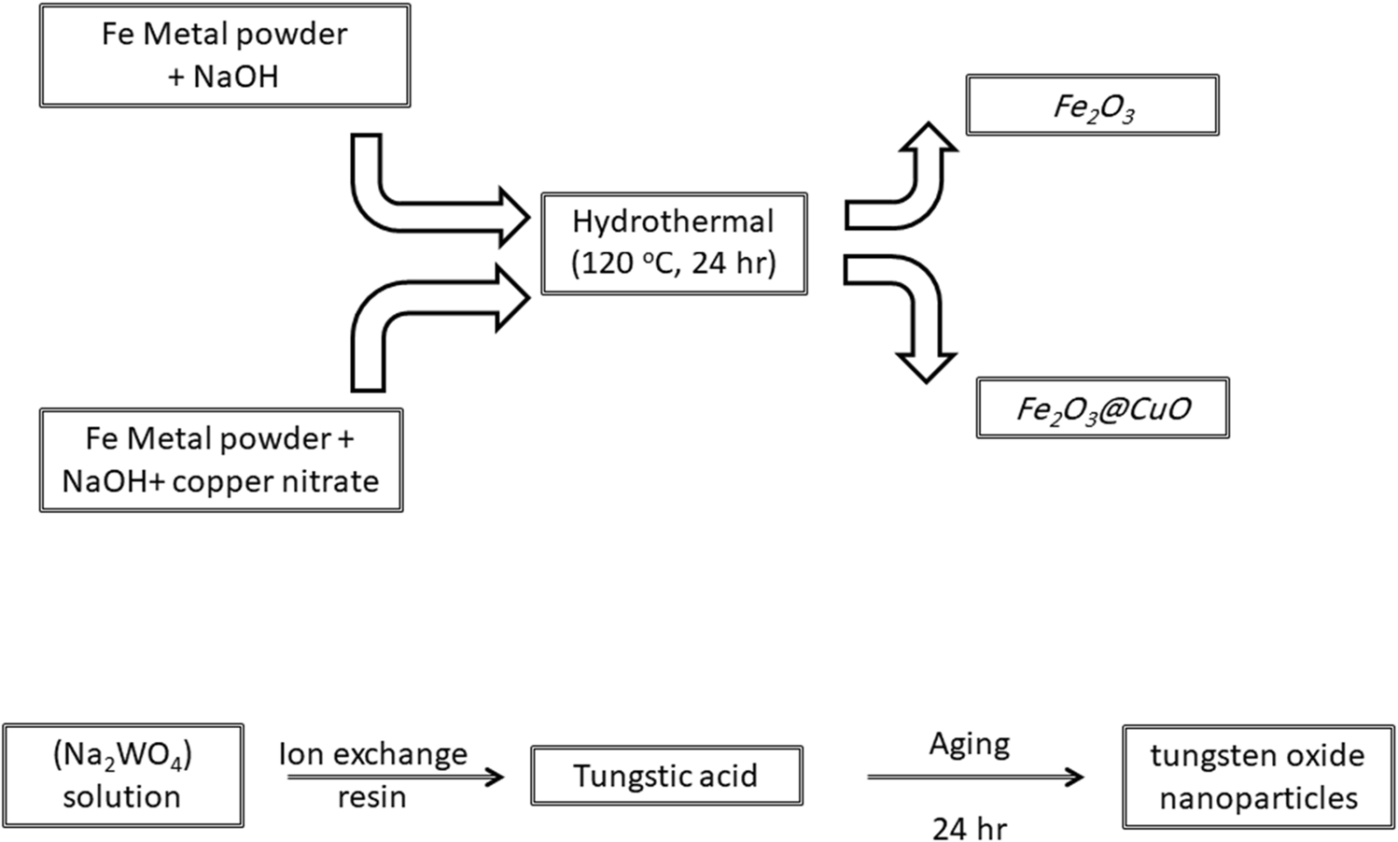
Schematic diagram showing in brief the synthesis procedure of Fe_2_O_3_, Fe_2_O_3_@CuO and WO_3_ NPs.

Magnetic Fe_2_O_3_ NPs were prepared via a one-pot hydrothermal reaction method as described elsewhere^45^. Typically, 4 g of iron metal powder was mixed with 10 g of NaOH in 40 mL of distilled water for 10 min at room temperature. The mixture was transferred into a Teflon-lined steel autoclave container and aged in an oven at 120 °C for 24 h. The obtained powder was washed several times with distilled water and dried overnight at 60°C (Scheme 1).

Cu-doped Fe_2_O_3_ (Fe_2_O_3_@CuO NPs) were prepared via one put hydrothermal reaction method as described elsewhere^45^. 4 g of iron metal powder was mixed with 10 g of NaOH in 40 ml of 0.1 M copper nitrate solution, where the water mixture was kept under harsh stirring for 10 min at ambient conditions. The mixture was transferred into a Teflon-lined steel autoclave container and aged in an oven at 120 °C for 24 h. The obtained powder was washed several times with distilled water and dried overnight at 60 °C.

Tungsten oxide (WO_3_ NPs) was synthesized as a previously reported method^45^. Briefly, 0.5 M Na_2_WO_4_ solution was prepared as described elsewhere as follows^45^: Sodium tungstate dihydrate (Na_2_WO_4_·2H_2_O, > 98%, Sisco, India) was dissolved in deionized milli-Q water. A column was packed with 30 ml of ion-exchange resin (Rohm& Haas, France). This column was washed several times with water before use. 10 ml of the (0.5 M Na_2_WO_4_) solution was loaded onto the column, to form yellowish and transparent tungstic acid (H_2_WO_4_) solution. The obtained solution was aged at room temperature for 24 h to produce precipitated tungsten oxide nanoparticles (Scheme 1).

### Characterization

The synthesized nanoparticles were characterized by several characterization tools. Scanning electron microscopy (SEM, JEOL, JSM-6360LA, Japan) was used to investigate the morphological structure of the obtained materials^46^. The crystallographic phases of the produced samples were determined by X-ray powder diffraction (XRD, Shimadzu-7000, Japan)^47^. Fourier transform infrared (FTIR) was used to perform the chemical structure of all compounds by A Bruker ALPHA spectrometer (Bruker Corporation, Rheinstetten, Germany).

### Biological activity evaluation

#### Antimicrobial activity

The antimicrobial activities of Fe_2_O_3,_ Fe_2_O_3_@CuO, and WO_3_ NPs were determined using the well-diffusion method as previously reported^48–50^. The bacteria and yeast cultures were grown in Luria–Bertani broth (0.5% yeast extract, 1% NaCl, and 1% tryptone) and Sabouraud dextrose broth (4% dextrose, 0.5% peptic digest of animal tissue, and 5% pancreatic digest of casein), respectively. The bacteria (10^6^ bacteria/ml) and yeast (10^4^ yeast/ml) were inoculated into 1% of the appropriate agar medium. After thoroughly shaking, 25 ml of the medium was transferred to sterile Petri plates (9 cm in diameter) and homogeneously distributed. Using a crock borer (6 mm in diameter), the wells were made into microbe agar plates^51^. The inhibitory concentration ranges were then determined by adding different concentrations of Fe_2_O_3,_ Fe_2_O_3_@CuO, and WO_3_ NPs (10, 50, 90, 130, 170, 210, 250, and 290 mg/ml) into these wells. Following that, varied concentrations of Fe_2_O_3_ NPs (5, 10, 15, and 20 mg/ml), Fe_2_O_3_@CuO NPs (10, 20, 30, and 40 mg/ml), and WO_3_ NPs (100, 150, 200, and 200 mg/ml) were loaded into the wells to determine the minimal inhibitory concentrations (MICs). Additionally, common antibiotics including 10 mg Ampicillin, 10 µg Penicillin, and 5 µg Ciprofloxacin discs were also surveyed as controls. The Petri dishes were then kept at 40 °C for an hour to allow the diffusion process to take place^52^. Then, the bacteria were incubated for 24 h at 37 °C and the yeasts for 72 h at 28 °C. Finally, the diameter of the created inhibitory zones on these plates was measured with a ruler (mm). After three repetitions of these experiments, average inhibition zones and their standard deviation values (mm ± SD) were calculated^53^.

#### MTT assay

Cytotoxicity of our synthesized NPS was determined by using MTT assay on *Vero* and *MCF-7* as normal models and human breast cancer cell lines, respectively^54^. Cells were seeded into a 96-well tissue culture plate with a density of (2 × 10^4^) cells/ml and then, incubated at ambient conditions (37 °C, 5% CO_2,_ and humidity of 85–95%) for 24 h until reached complete sheet^55^. Afterward, cells were treated with synthesized NPs (Fe_2_O_3_, Fe_2_O_3_@CuO, and WO3 NPS) with concentrations (100, 50, 25, 12.5, 6.25, 3.125 µg/ml) for 48 h. Cell viability (%) was determined by applying MTT dye for 4 h then, 100 µl of DMSO was added to dissolve the formed crystals. The OD was measured at 570 nm using a microplate reader (CLARIOstar Plus, BMG LABTECH, Germany)^56,57^.

1 $$Cell \,viability \,\left(\mathrm{\%}\right)= \frac{Mean \,OD \,(S)}{Mean \,OD \,(C)}\times 100$$

Where OD (S) is the mean optical density of the tested sample and OD (C) refers to the mean optical density of the control group^58^. The relative cell viability % was plotted against the concentrations of the prepared NPs using *GraphPad Prism Version 6*.

#### Antiviral assay against human adenovirus type 5 (ADV-5)

##### Virucidal mechanism

*Vero* cells were seeded into a 6-well tissue culture plate with a density of (5 × 10^5^ cells/well) till reached 90% confluency after 24 h incubation. Then, the cells were treated with synthesized materials (Fe_2_O_3_, Fe_2_O_3_@CuO, and WO_3_ NPs) after their incubation at 4 °C for 1 h with 100 TCID50 of ADV load^59^. This procedure is based on the ability of the tested material to interact with the virus preventing its ability to replicate inside the host cells^60,61^. Then, viral copies were quantified by quantitative real-time PCR (RT-PCR)^62^.

##### Viral adsorption mechanism

The viral adsorption mechanism was done via seeding *Vero* cells into a 6-well tissue culture plate at a density of (5 × 10^5^) cells/well, then incubated for 24 h at ambient conditions. Upon reaching a confluency of > 90%, cells were treated with the tested NPs, then incubated for another 24 h. On the third day^63^, the cells were infected with 100 TCID50 of the virus until the appearance of cytopathogenic effect (CPE)^64^. Finally, viral load was determined by quantitative RT-PCR^60,65^. The mechanism relies on the ability of tested material to inhibit viral entry into cells.

### Statistical analysis

The obtained data were statically analyzed using an unpaired t-test with GraphPad Prism. The values were presented as the mean ± SD.

### Ethics approval and consent to participate

Manuscripts report no studies involving human participants, human data, or human tissue. All experiments were performed in accordance with the Guidelines of the World Medical Association Declaration of Helsinki: Ethical Principles for Medical Research Involving Human Subjects and approved by the ethics committee at Cairo University and The British University in Egypt (BUE).

## Results and discussion

### Crystal structures investigation by XRD analysis

**Figure 1:**
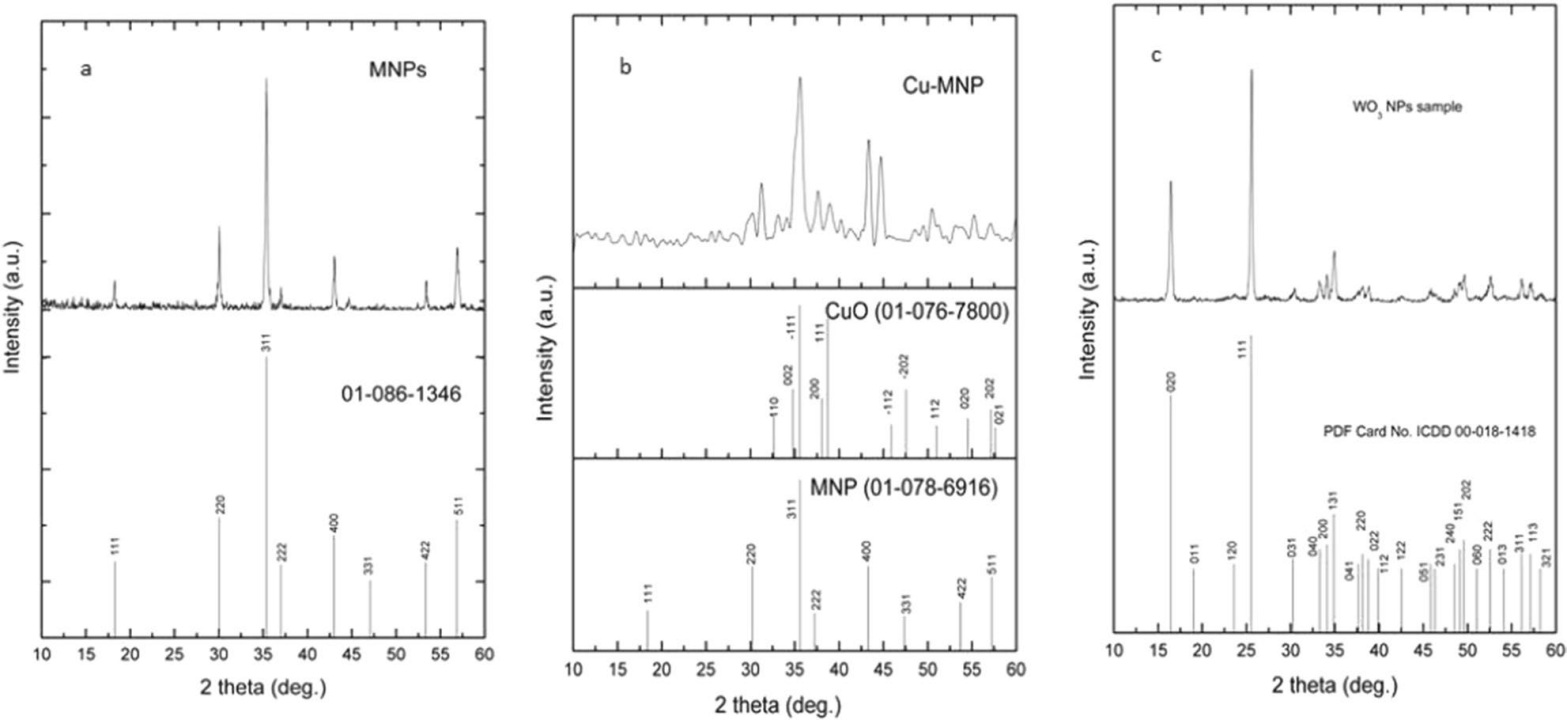
XRD pattern of synthesized magnetic NPs and their matched patterns as Fe_2_O_3_, Fe_2_O_3_@CuO and WO_3_ (**a**–**c**); respectively.

Figure 1 shows the XRD patterns of all synthesized magnetic nanoparticles (*i.e.* Fe_2_O_3_, Fe_2_O_3_@CuO, and WO_3_ MNPs). XRD patterns show a single-phase structure of formed Fe_2_O_3_; while all peaks were indexed to the cubic Fe_3_O_4_ with a space group of Fd-3m (227) and lattice parameters: 8.3560 Å (ICDD Card No. 01-078-6916) (Fig. 1a).

Figure 1b shows the XRD pattern of synthesized Cu doped-MNPs. XRD pattern of Fe_2_O_3_@CuO shows a dual phase; where all peaks were indexed to the cubic Fe_3_O_4_ with a space group of Fd-3m (227) and lattice parameters: 8.3560 Å (*ICDD Card No. 01-078-6916*) and monoclinic CuO with a space group of C2/c (15) and lattice parameters: a ~ 4.7940 Å, b ~ 3.3620 Å, c ~ 5.2280 Å (*ICDD Card No. 01-076-7800*) (Fig. 1b). The obtained pattern is further evidencing the formation of dual phase between CuO doped onto Fe_2_O_3_.

Figure 1c shows the XRD pattern of synthesized tungsten oxide nanoparticles (WO_3_ NPs). XRD pattern of tungsten oxide nanoparticles show a single phase, where all peaks were indexed to the orthorhombic WO_3_·H_2_O with a space group of Pmnb (62) and lattice parameters: a ~ 5.2477 Å, b ~ 10.7851 Å, c ~ 5.1440 Å (*ICDD Card No. 00-018-1418*). The obtained patterns of WO_3_ NPs are fully consistent with the previously published findings of Elnouby et al.^45^.

The crystal sizes of the obtained nanoparticles were calculated from Debye-Scherer equation^42^:

2 $$D = \frac{K\lambda }{{\beta \cos \theta }}$$

where λ = 0.1542 nm is the Cu-K_α_ wavelength, *K* is a constant and is the FWHM.

Table 1 summarizes the crystal sizes of the obtained materials. It is noticeable that all obtained materials are in nanoscale size. Pure octahedral MNP has a crystal size of 35 nm, and by adding Copper the crystal size decreased to 7 nm coinciding with the disappearance of octahedral structure (Fig. 3). While tungsten oxide platelets have a lateral dimension of 33 nm and thickness of 25 nm.

**Table 1:** Crystal sizes of the obtained synthesized materials.

Sample type	2Ɵ	Plane	Size (nm)
Fe_2_O_3_	35.37	311	35.5
Fe_2_O_3_@CuO	35.61	MNP (311) Cu (-111)	7
WO_3_	25.61	111	33.5
	16.45	020	25.5

### FT-IR analysis

**Figure 2:**
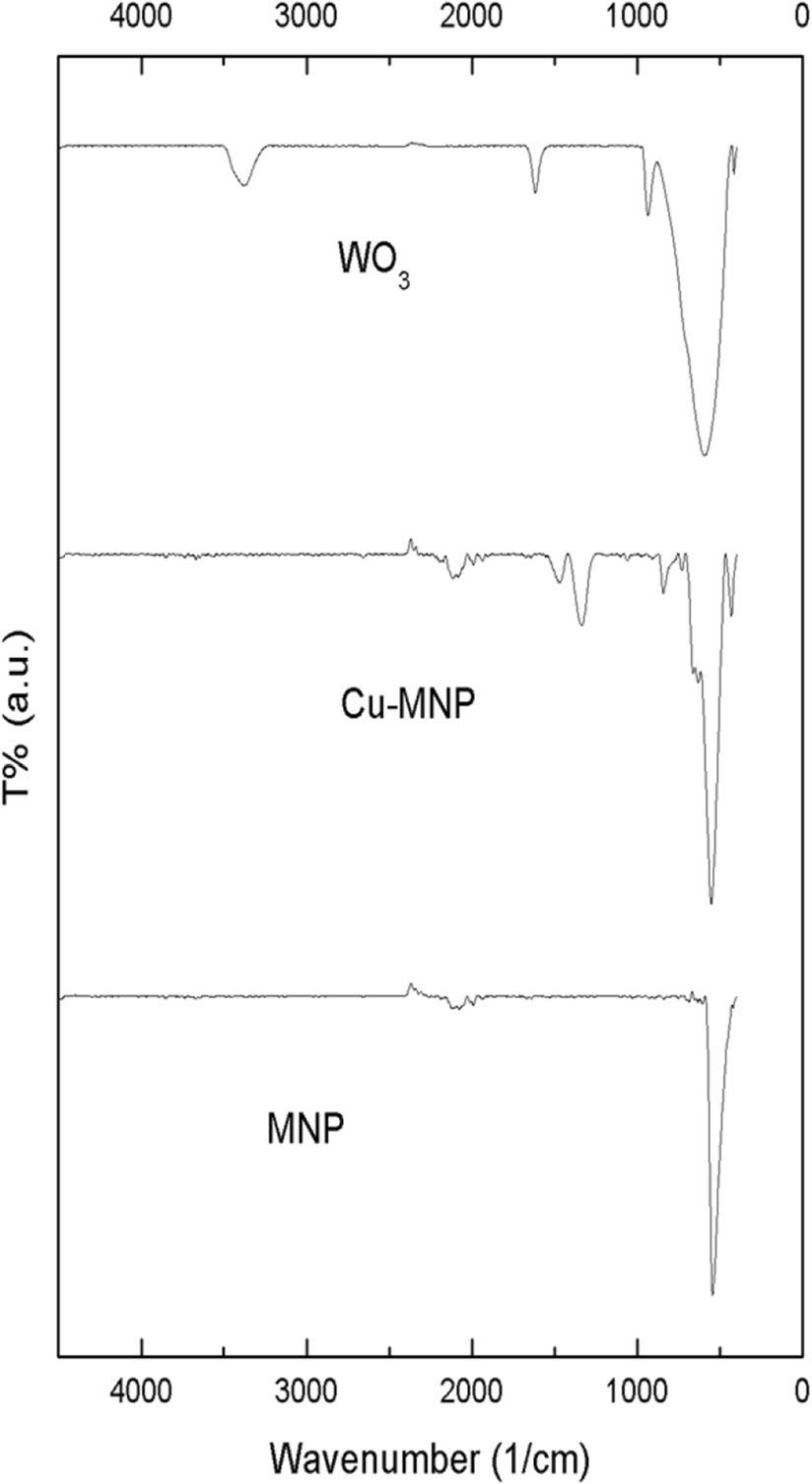
FT-IR spectra of synthesized magnetic NPs as Fe_2_O_3_, Fe_2_O_3_@CuO and WO_3_ (down-to-up); respectively.

Figure 2 shows FTIR spectra of all synthesized Fe_2_O_3_, Fe_2_O_3_@CuO, and WO_3_ MNPs. It was observed that the FTIR spectrum of Fe_2_O_3_ shows characteristics peaks at ν 3406 cm^−1^ revealing the stretching motion of (O–H) and the medium narrow band at ν 1616 cm^−1^, characteristic of in-plane bending of (H–O-H) of the water molecule. Very intense broadband in the region at ν 902–621 cm^−1^ corresponds to different motions arising from W–O linkage^66^. Therefore, the band at ν 902 cm^−1^ refers to stretching (W = O_t_) (where O_t_ is the terminal oxygen). While the bands at ν 763 and 694 cm^−1^ revealed the stretching (W–O) and the band at ν 713 cm^−1^ is due to stretching (W–O-W)^67^. On the other hand, the FTIR spectrum of synthesized Fe_2_O_3_ MNPs shows characteristic peaks also at the broadband at around 624–425 cm^−1^ revealing the vibrations of the Fe–O bonds of the magnetite^68^.

Similarly, the FTIR spectrum of prepared Cu-doped MNP shows characteristic peaks at ν 525 cm^−1^ revealing the bending vibration of the Cu–O bond^69^. Briefly, all synthesized magnetic metal oxides (*i.e.* Fe_2_O_3_, Fe_2_O_3_@CuO, and WO_3_ MNPs) were characterized by a broad band around at ν 500 cm^−1^ indicating different modes of bending vibration of the metal–O bond. In addition, a few individual characteristic peaks are presented, where these peaks result from the crystal structures of nanoparticles, which play a definite role in their performance.

### SEM investigation

**Figure 3:**
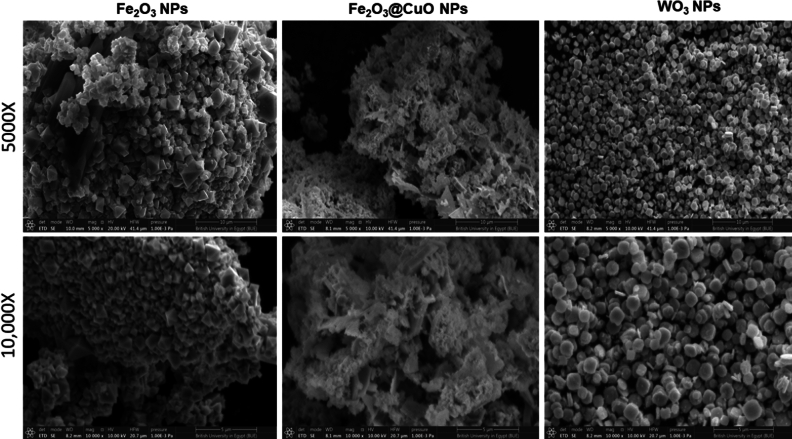
SEM micrographs of synthesized magnetic NPs of Fe_2_O_3_, Fe_2_O_3_@CuO and WO_3_, where all images were taken at (original magnification 5000X and 10,000X, scale 10 and 5 µm and applied voltage at 20 kV).

Figure 3 shows the SEM surface investigation of all synthesized MNPs at two original magnifications. It was observed that Fe_2_O_3_ MNPs have a uniform octahedral structure and size. After adding CuO into Fe_2_O_3_MNPs, it lost its uniform octahedral structure, leading to forming of a homogeneously coated bilayer structure that uniformly distributed on the surface of the Fe_2_O_3_ MNPs. Notably, an SEM micrograph revealed that WO_3_ NPs composed of a large number of square nano-plates. In addition, the average particle size of Fe_2_O_3_ and WO_3_ NPS was measured from SEM micrographs. Size distribution histograms were listed supplementary in Fig. S1 (*supplementary data*). The average size of octahedral MNPs was 1.5 ~ 2.0 µm. However, the lateral dimension of the WO_3_ nano-plates reached several hundreds of nanometers.

### EDAX analysis

**Figure 4:**
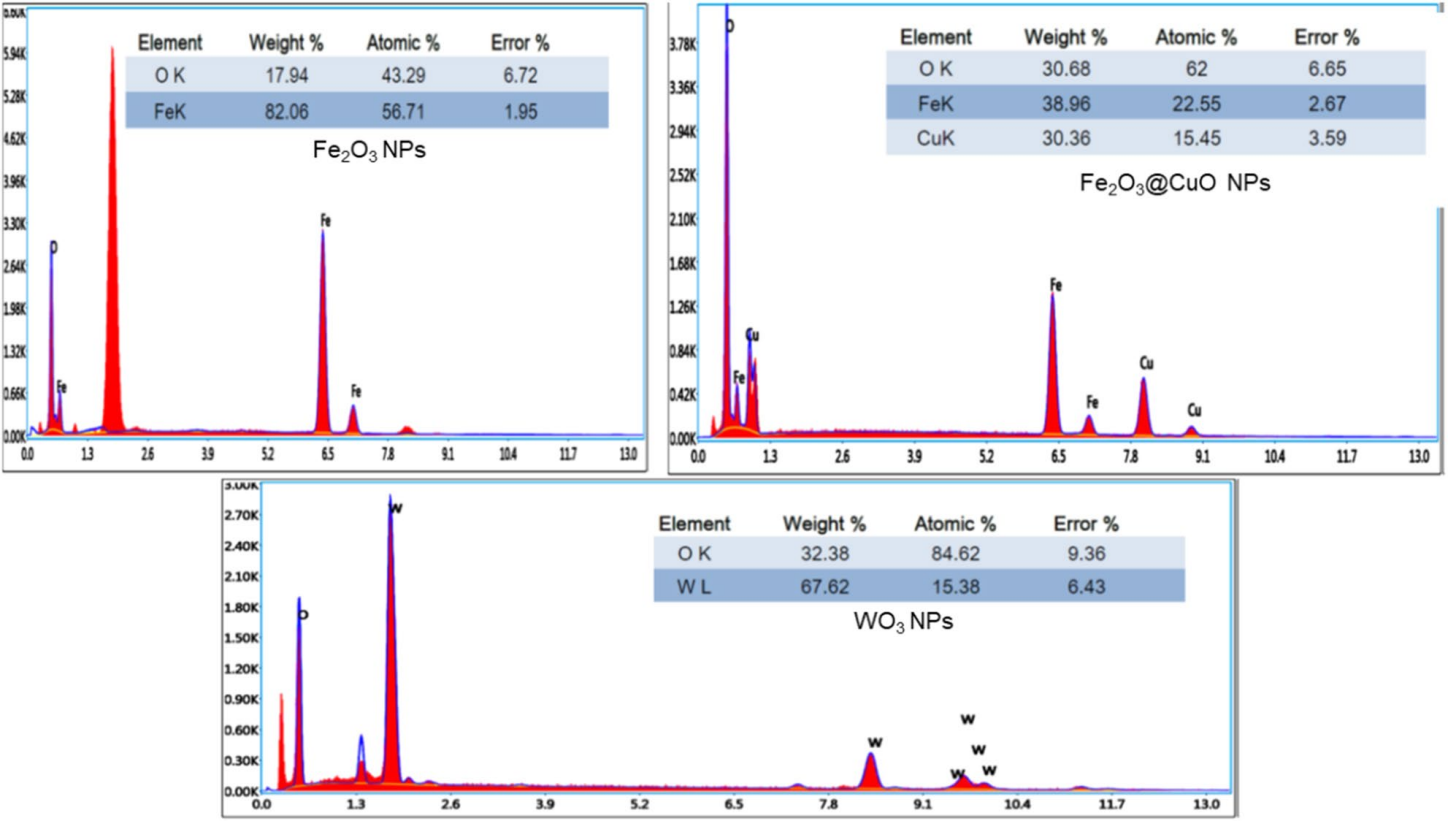
EDAX analysis of synthesized magnetic NPs of Fe_2_O_3_, Fe_2_O_3_@CuO and WO_3_.

The compositional and elemental analysis of all synthesized MNPs was examined and verified using an EDAX-SEM unit and shown in Fig. 4. The composition of Fe_2_O_3,_ Fe_2_O_3_@CuO, and WO_3_ NPs were (Fe, O), (Fe, O, Cu), and (W, O); respectively, which indicate that no contaminated elements were detected for all samples. In additions, the composition ratios in all provided samples confirmed its compositions- as listed in Table 2 from EDX analysis. These results are in good agreement with the crystal structures provided by XRD investigations (Figs. 1 and 2).

**Table 2:** EDX results of the prepared NPs samples.

Fe_2_O_3_		Fe_2_O_3_@CuO		WO_3_	
Element	Wight (%)	Element	Wight (%)	Element	Wight (%)
O	17.94	O	30.68	O	84.62
Fe	82.06	Fe	38.96	W	15.38
		Cu	30.36		
Total	100	Total	100	Total	100

### Biological activity evaluations

#### Antimicrobial activity

**Figure 5:**
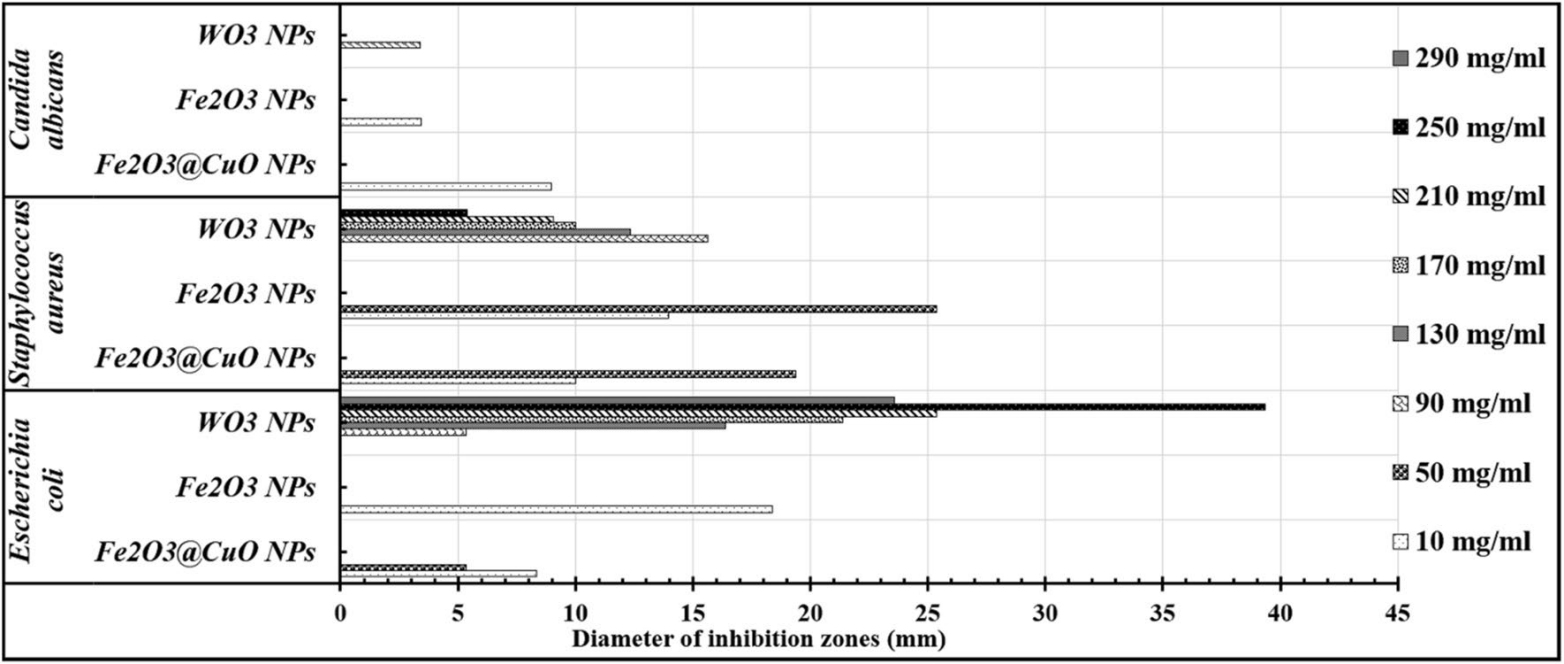
Survey of the inhibitory concentration ranges of Fe_2_O_3_@CuO NPs, Fe_2_O_3_ NPs, and WO_3_ NPs against some human pathogens including *E. coli*, *S. aureus*, and *C. albicans.*

To evaluate the antimicrobial efficacy, synthesized Fe_2_O_3,_ Fe_2_O_3_@CuO, and WO_3_ NPs were studied individually against different human pathogens such as *E. coli, S. aureus,* and *C. Albicans*. A wide range of tested nanomaterial concentrations (10, 50, 90, 130, 170, 210, 250, and 290 mg/ml) were studied to find appropriate ranges for all examined nanoparticles. According to Fig. 5, the highest inhibition zones of Fe_2_O_3_@CuO NPs and Fe_2_O_3_ NPs were observed at low concentrations that ranged from (10 to 50 mg/ml). Nevertheless, the largest concentrations of WO_3_ NPs (90 to 250 mg/ml) produced the widest inhibition zones against all tested pathogens, as shown in (Fig. 5). Also, *C. Albicans* showed clear resistance nearly to all tested nanoparticles; whereas *Gram*-negative bacteria are affected perfectly, followed by *Gram*-positive bacteria.

**Figure 6:**
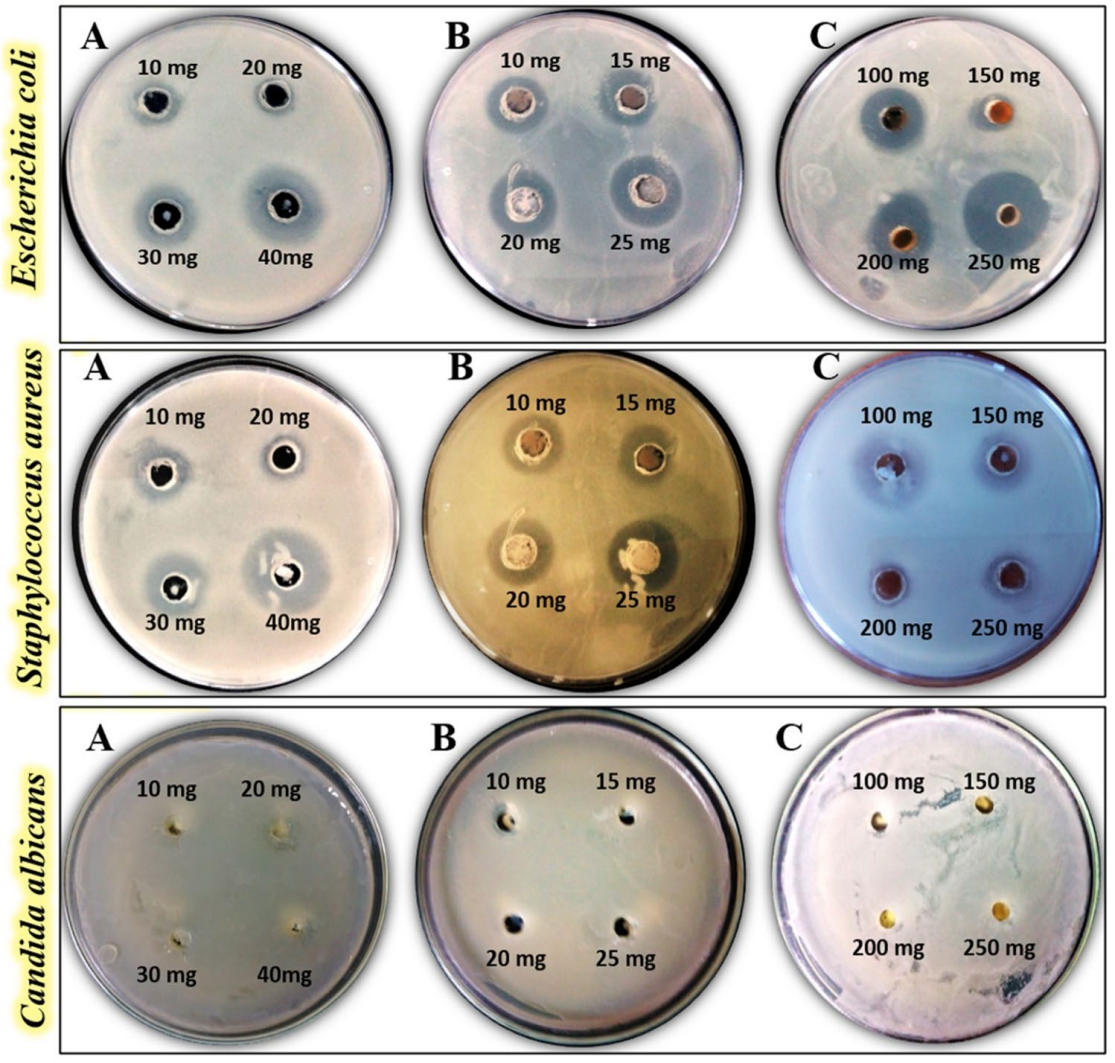
Antimicrobial activity of Fe_2_O_3_@CuO NPs (**A**); Fe_2_O_3_ NPs (**B**) and WO_3_ NPs (**C**) against tested human pathogens*.*

**Figure 7:**
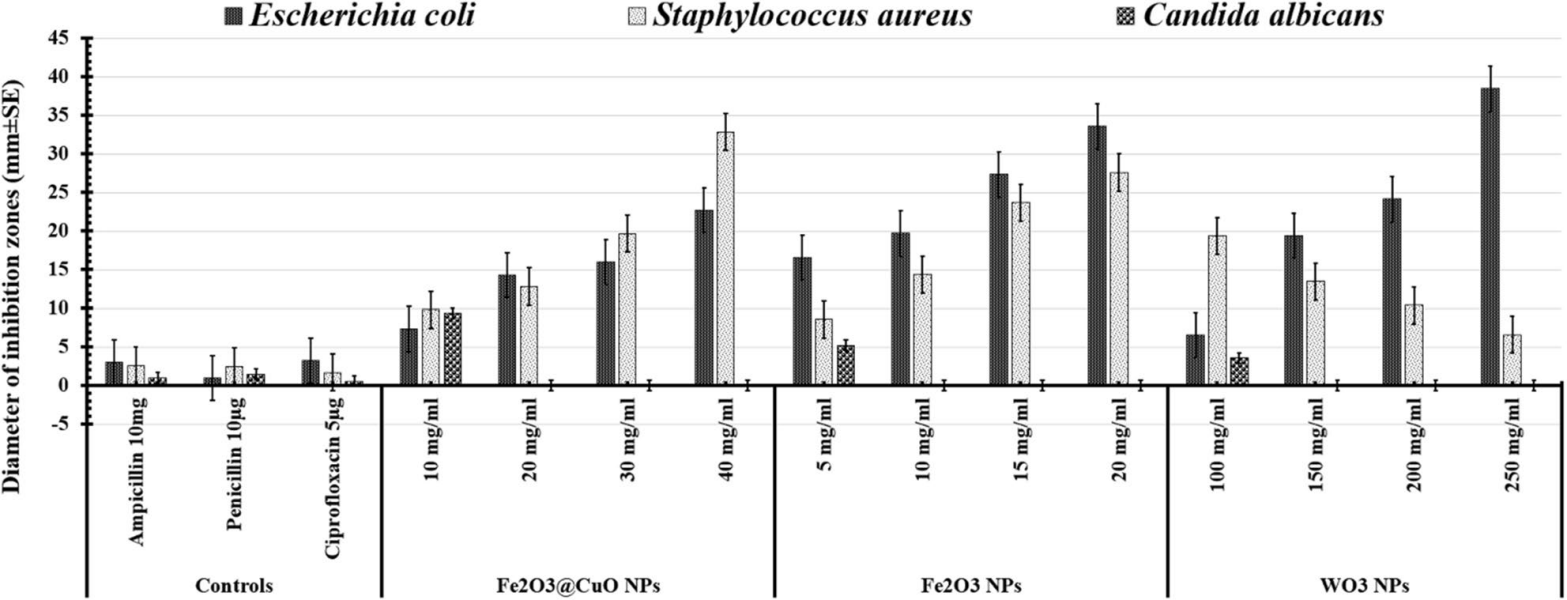
Antimicrobial activity of Fe_2_O_3_@CuO NPs (10 mg/ml**,** 20 mg/ml**,** 30 mg/ml, and 40 mg/ml)**;** Fe_2_O_3_ NPs (10 mg/ml, 15 mg/ml, 20 mg/ml, and 25 mg/ml), and WO_3_ NPs (100 mg/ml**,** 150 mg/ml, 200 mg/ml, and 250 mg/ml) against different human pathogens compared to various conventional antibiotics as controls, including 5 µg of Ciprofloxacin, 10 µg of Penicillin, and 10 mg of Ampicillin.

Subsequently, the MICs for all tested nanoparticles were determined as shown in Table 3. The inhibition zones generated by applying different doses of Fe_2_O_3_@CuO, Fe_2_O_3_, and WO_3_ NPs against all tested human pathogens are depicted in the antimicrobial photographs in Fig. 6. When compared to controls, all of the synthesized Fe_2_O_3_, Fe_2_O_3_@CuO, and WO_3_ NPs exhibit significant antimicrobial effects against all tested human pathogens. It was clearly observed that the highest antibacterial potency was detected against *E. coli* (38.45 ± 3.12 mm) in case of WO_3_ NPs at 250 mg/ml, followed by 20 mg/ml of Fe_2_O_3_ NPs (33.56 ± 3.25 mm). However, the lowest inhibition zone was determined in case of *E. coli* (22.67 ± 2.08 mm) at 40 mg/ml of Fe_2_O_3_@CuO NPs (Table 3 and Fig. 7). However, in the case of *S. aureus*, the maximum antibacterial potency was recorded at 40 mg/ml of Fe_2_O_3_@CuO NPS (32.86 ± 3.21 mm) and the lowest one was recorded at 100 mg/ml of WO_3_NPs (19.36 ± 1.08 mm), as shown in (Fig. 7). This indicates that antimicrobial activity depends on both type of bacterial species and nanoparticles concentrations. Nevertheless, the resistance was relatively dominant for all tested nanoparticles (Fe_2_O_3_@CuO, Fe_2_O_3_, and WO_3_ NPs) in the case of fungal cells (Table 3 and Fig. 7).

**Table 3:** Antimicrobial activity of different concentrations of Fe_2_O_3_, Fe_2_O_3_@CuO and WO_3_ NPs against varied human pathogens compared with different common antibiotics as controls.

Treatments	Concentrations	*Escherichia coli*	*Staphylococcus aureus*	*Candida albicans*
Controls	Ampicillin 10 mg	3.04 ± 0.02	2.54 ± 0.14	1.02 ± 0.98
	Penicillin 10 µg	0.96 ± 1.02	2.48 ± 0.39	1.45 ± 0.92
	Ciprofloxacin 5 µg	3.21 ± 1.98	1.69 ± 0.95	0.54 ± 0.95
Fe2O3@CuO NPs	10 mg/ml	7.3 ± 3.61	9.8 ± 4.63	9.36 ± 2.36
	20 mg/ml	14.26 ± 1.52	12.79 ± 4.56	0
	30 mg/ml	16 ± 3.65	19.67 ± 7.78	0
	40 mg/ml	22.67 ± 2.08	32.86 ± 3.21	0
Fe_2_O_3_ NPs	5 mg/ml	16.56 ± 1.53	8.56 ± 0.69	5.23 ± 0.59
	10 mg/ml	19.68 ± 4.04	14.36 ± 2.23	0
	15 mg/ml	27.3 ± 7.09	23.67 ± 1.36	0
	20 mg/ml	33.56 ± 3.25	27.58 ± 3.58	0
WO_3_ NPs	100 mg/ml	6.56 ± 5.51	19.36 ± 1.08	3.56 ± 0.14
	150 mg/ml	19.36 ± 4.35	13.45 ± 0.96	0
	200 mg/ml	24.12 ± 3.06	10.36 ± 0.96	0
	250 mg/ml	38.45 ± 3.12	6.59 ± 0.89	0

Recently, polymers modified with large-surface-area of NPs, such as Fe_2_O_3_, and WO_3_ NPs have been employed for a variety of applications including drug release, tissue regeneration, heavy metal adsorption, cell separation, antimicrobial agents, and the treatment of malignant brain tumours and breast cancer cells^70,71^. They have low volume/surface area ratios, high adsorption capabilities, and selective target molecule adsorption^72^. While, another report has revealed that the charge potential of both the fabricated nanoparticles and the tested microbial cells influences antimicrobial properties. Furthermore, the Concentration, shape, and size of the nanoparticles generated have an impact on overall bioactivity, which are among the primary causes for NP attachment or non-attachment to microbial cells.

According to Pekdemir et al.^73^, Fe_2_O_3_NP-pathogen cell contact would be poor due to prevailing electrostatic repulsion at the interface, which is the underlying cause of the NP's non-attachment to the microbial cells. Moreover, at high concentrations of at least 50 µM (critical concentration); they observed some antimicrobial effects. Also, Borcherding et al.^74^, reported excellent antimicrobial activities at different n-IONP dosages, which are consistent with our findings. Since increased n-IONP concentrations in the culture media might also promote the production of reactive oxygen species (ROS) (including superoxide radical, hydrogen peroxide, and hydroxyl radical), which is one of the key causes of metal oxide nanoparticles' antimicrobial properties^75,76^. Furthermore, physical contact between nanoparticles and microbial wall membranes modifies cell permeability, which subsequently leads to microbial mortality^77,78^.

#### Cytotoxicity test

##### MTT-assay

**Figure 8:**
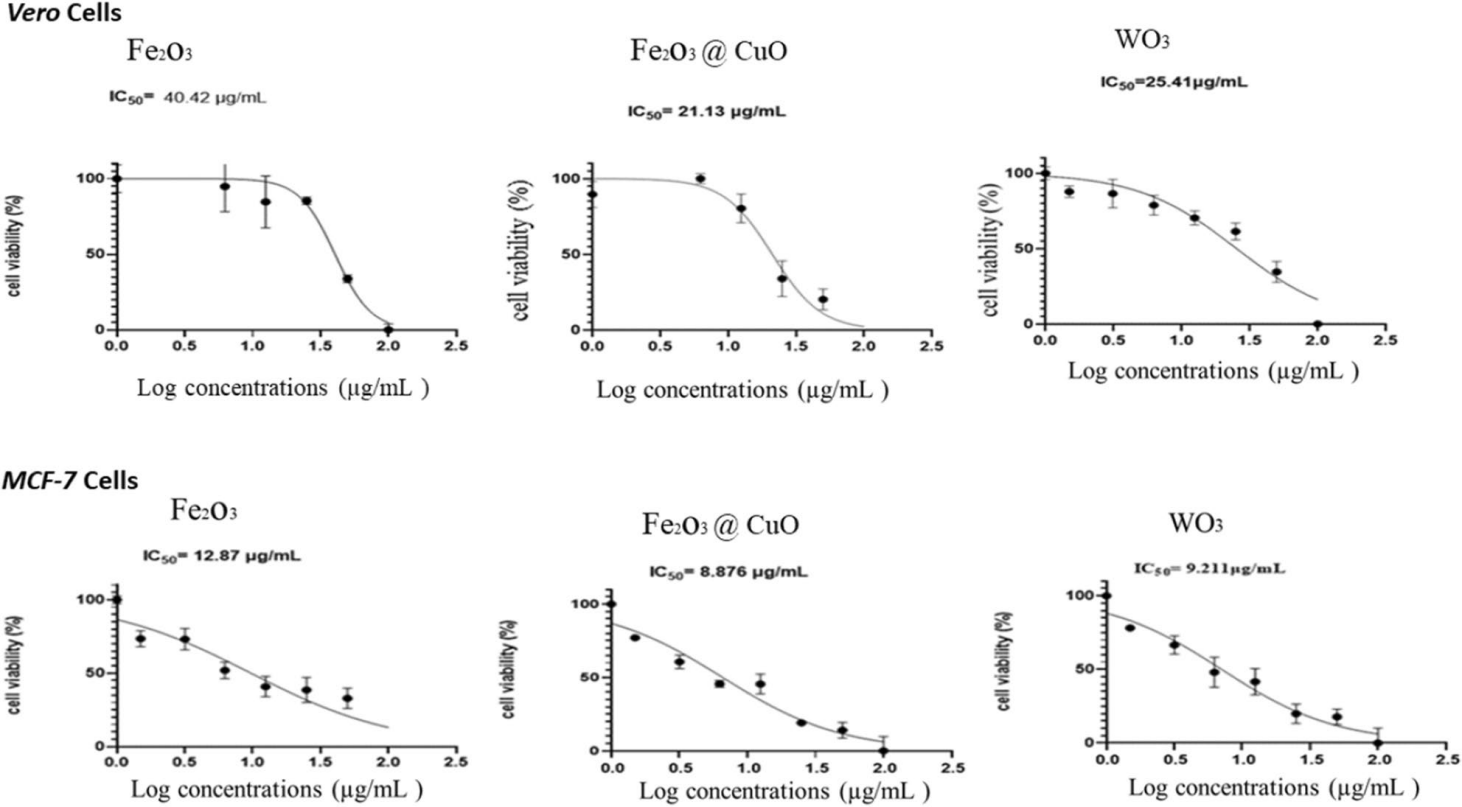
IC_50_ charts through MTT-assay of synthesized Fe_2_O_3_, Fe_2_O_3_@CuO and WO_3_ NPs with different concentrations using *Vero* (normal cell line) (up) and *MCF-7* (cancer cell line) (down).

MTT colorimetric assay was performed on *Vero* and *MCF-7* cell lines with serial concentrations of each synthesized NPs, as shown in Fig. 8. After treatment with different concentrations of NPs, the results were obtained by calculation of IC50 value using *Graph Pad Prism software version 6*.

IC50 values were detected at concentrations of 40.24, 21.13, and 25.41 µg/ml for Fe_2_O_3_, Fe_2_O_3_@CuO, and WO_3_NPs respectively on Vero cells, whereas on MCF-7 these values were detected at 12.87, 8.876 and 9.211 µg/ml; respectively for the same materials. Our tested NPs demonstrated anti-proliferative activity against replication of in vitro model of human breast cancer cells (MCF-7) (Fig. 8).

Furthermore, morphological examination of *MCF-7* cells revealed a distinctive change in the morphology of *MCF-7* cells from a healthy spindle-like shape to a rounded morphology, and cells were also observed to form small irregular aggregations due to the toxicity induced by magnetic NPs on them. Such toxicity is due to the production of reactive oxygen species (ROS)^19,37^ DNA injury and fragmentation, lipid peroxidation and genotoxic effect through chromosomal damage^27^. Leading to caspase activation which push cancer cells apoptotic pathway^79^ and cell death in response to the toxic effect of magnetic nanoparticles. These findings support the anti-cancer effects of the synthesized magnetic NPs^22,80^.

#### Antiviral assay against human Adenovirus type-5(ADV-5) on Vero cells

Quantitative measurement of antiviral activity using real-time PCR against Adenovirus (ADV-5) was determined for our proposed materials using two mechanisms including virucidal and viral adsorption mechanisms. Results showed that all tested materials have antiviral activity via viral adsorption mechanism for both Fe_2_O_3_@CuO and WO_3_ NPs as evidenced by the undetectable level of viral load (copies/mL) although, Fe_2_O_3_ NPs decreased the viral copies reach 80% reduction compared with the positive control. This indicates that the nanoparticles' effect was to prevent viruses' entry into host cells^61,81^.

##### Virucidal mechanism

**Figure 9:**
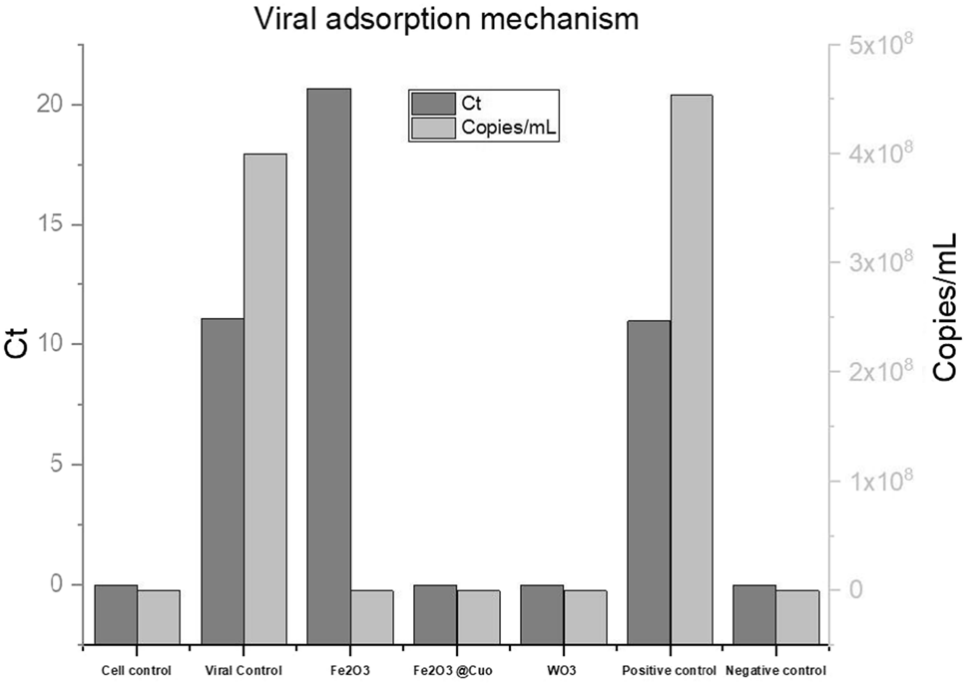
Chart of viral adsorption mechanism represented against Adino virus.

Figure 9 represents a chart of viral adsorption mechanism. This mechanism depended on investigating viral titer through the ability of the tested nanoparticles to neutralize the virus and block its affinity to enter and infect the cells Therefore, lost its ability to replicate inside the cells^82^ Results showed the synthesized Fe_2_O_3_, Fe_2_O_3_@CuO, and WO_3_ NPs inhibited the viral titer as evidenced by this assay (Table 4). The viral titer was involved (copies/mL) as a result the tested materials showed high antiviral properties against *ADV-*5 and detected levels of viral copies using quantitative real-time PCR (Table 4). Fe_2_O_3_ treated infected cells gave viral titer (3.3 × 10^4^) Copies/ml, Fe_2_O_3_@Cuo treated infected cells was (2 × 10^5^ Copies/ml), WO3 treated infected cells was (8.5 × 10^4^ Copies/ml), showing the reduction in viral titer as 99% reduction compared to viral control (4.54 × 10^8^ Copies/ml). Consequently, this detailed study as virucidal and viral adsorption mechanisms of MNPs proved the high affinity of magnetic NPs as a potent antiviral agent^40^.

**Table 4:** Real-time PCR assay of AdV-5 treated cells with synthesized MNPs using (virucidal mechanism).

AdV-5	CT	Copies/mL
Untreated *Vero* cells (cell control)	Under detection	Under detection
Positive cells infected with AdV5 (viral control)	11.08	4 × 10^8^
Fe_2_O_3_-treated infected cells	19.16	3.3 × 10^4^
Fe_2_O_3_@Cuo NPs treated infected cells	19.69	2.0 × 10^5^
WO3-treated infected cells	20.71	8.5 × 10^4^
Positive control	10.97	4.54 × 10^8^
Negative control	Under detection	Under detection

##### Viral adsorption mechanism

Concerning the antiviral adsorption mechanism this mechanism depended on the affinity of the tested materials to inhibit the viral entry, and replication and also prevent its spread to cells during its pathogenesis process, two materials were able to prevent viral replication inside the cells, thus demonstrating the antiviral activity without affecting cell viability (Table 5). The results demonstrated the potent of Fe_2_O_3_ as an antiviral inhibitor by decreasing the viral titer and rationalized their effect an equivalent reduction of viral titer reach to 99% compared to the positive control, the viral titer for Fe_2_O_3_ NPs was (8.8 × 10^4^), compared to the positive control was 4.54 × 10^8^ while Fe_2_O_3_@CuO and WO_3_ NPs showed the great effect that the viral load was under detectable inside the treated cells. MNPs cause irreversible damage to the viral genome and inactivation of viral genome replication which prevents viral replication inside the treated cells. By another mechanism, MNPs block the viral entry into the cells, as evidenced by undetected viral copies by real-time PCR assay^83,61^.

**Table 5:** Real-time PCR assay of AdV-5 treated cells with synthesized MNPs using (Viral adsorption mechanism).

AdV-5	CT	Copies/ml
Untreated Vero cells (cell control)	Under detection	Under detection
Positive cells infected with AdV5 (viral control)	11.08	4 × 10^8^
Fe_2_O_3_ treated infected cells	20.68	8.8 × 10^4^
Fe_2_O_3_ @Cuo treated infected cells	Under detection	Under detection
WO3-treated infected cells	Under detection	Under detection
Positive control	10.97	4.54 × 10^8^
Negative control	Under detection	Under detection

## Conclusions

In conclusion, Fe_2_O_3_, Fe_2_O_3_@CuO, and WO_3_ NPs were successfully synthesized, fully characterized, and structure evaluated; these materials were greatly applied in biomedical aspects due to their biocompatibility and chemical stability which play a great role as an antiproliferative effect against breast cancer, IC50 on MCF-7(human breast cancer) was detected at 12.87, 8.876, and 9.211 µg/ml for Fe_2_O_3,_ Fe_2_O_3_@CuO, and WO_3_ respectively. The combination of iron oxide with copper oxide improved the anti-proliferative activity of iron oxide and increased its toxicity against the replication of cancer cells. The result showed that iron oxide modified with copper oxide nanoparticles (Fe_2_O_3_@CuO NPs) demonstrated the highest anticancer activity against an in vitro model of human breast cancer cells affecting their morphological appearance and confirmed by low IC50 value. Fe_2_O_3,_ Fe_2_O_3_@CuO, and WO_3_ were observed against replication of human adenovirus type 5 as a respiratory viral model. Fe_2_O_3_@CuO and WO_3_ gave a complete reduction of viral titer to an undetectable level, and Fe_2_O_3_ gave a reduction of more than 60% which proves the high potency of these tested materials against viral infection. In addition, their activity was demonstrated against gram-positive and gram-negative bacteria. In this study, we obtained a clear view of the potency of the tested MNPs as promising antiviral agents against ADV-5 (respiratory viral model). Therefore, our synthesized materials, Fe_2_O_3_, Fe_2_O_3_@CuO, and WO3 NPs, showed promising candidates and will be subjected to further insight and investigation on a molecular level to elucidate the interplay between apoptotic gene expression (programmed cell death) and their role in cancer death and in vivo studies before their application in clinical settings.

### Supplementary Information

Supplementary Information.

## Data Availability

The datasets used and/or analysed during the current study available from the corresponding author on reasonable request.
