# Supplementary material for: Facile synthesis of Fe2O3, Fe2O3@CuO and WO3 nanoparticles: characterization, structure determination and evaluation of their biological activity
Source: Sci Rep. 2024 Mar 13;14:6081. doi: 10.1038/s41598-024-55319-8 (PMC10937632; doi:10.1038/s41598-024-55319-8)
Supplement: Supplementary file 1 — Supplementary Information. [file 41598_2024_55319_MOESM1_ESM.docx]

Facile synthesis of Fe_2_O_3_, Fe_2_O_3_@CuO and WO_3_ nanoparticles: Characterization, structure determination and evaluation of their biological activity

Asmaa T. Mohamed^1^, Reda Abdel Hameed^2,3^ , Shahira H. EL-Moslamy^4^, , Mohamed Fareid^2,3^, Mohamad othman^2,3^, Samah A. Loutfy^1,5^, Elbadawy A. Kamoun^1,6*^ Mohamed Elnouby^7*^

^1^Nanotechnology Research Center (NTRC), The British University in Egypt, El-Shorouk City, Suez Desert Road, Cairo 11837, P.O. Box 43, Egypt.

^2^Basic Science Department, Preparatory Year, University of Ha’il, 1560, Hail, KSA.

^3^Medical and Diagnostic Research Centre, University of Ha'il, Ha'il 55473, Saudi Arabia.

^4^Bioprocess Development Dep., Genetic Engineering and Biotechnology Research Institute (GEBRI), City of Scientific Research and Technological Applications (SRTA-City), New Borg Al-Arab City, Alexandria 21934, Egypt.

^5^Virology and Immunology Unit, Cancer Biology Dep., National Cancer Institute (NCI), Cairo University, Fom El-Khalig 11796, Cairo, Egypt.

^6^Polymeric Materials Research Dep., Advanced Technology and New Materials Research Institute (ATNMRI), City of Scientific Research and Technological Applications, New Borg Al-Arab City 21934, Alexandria, Egypt.

^7^Nanotechnology and Composite Materials Dep., Advanced Technology and New Materials Research (ATNMRI), City of Scientific Research and Technological Applications (SRTA-City), New Borg Al-Arab City, Alexandria 21934, Egypt.

******Corresponding author****: Mohamed Elnouby, E-mail:* [*m_nano2050@yahoo.com*](mailto:m_nano2050@yahoo.com)*; and E.A. Kamoun, E-mail: elbadawy.kamoun@bue.edu.eg,* [*badawykamoun@yahoo.com*](mailto:badawykamoun@yahoo.com)*.*

*3.3. SEM investigation*

*
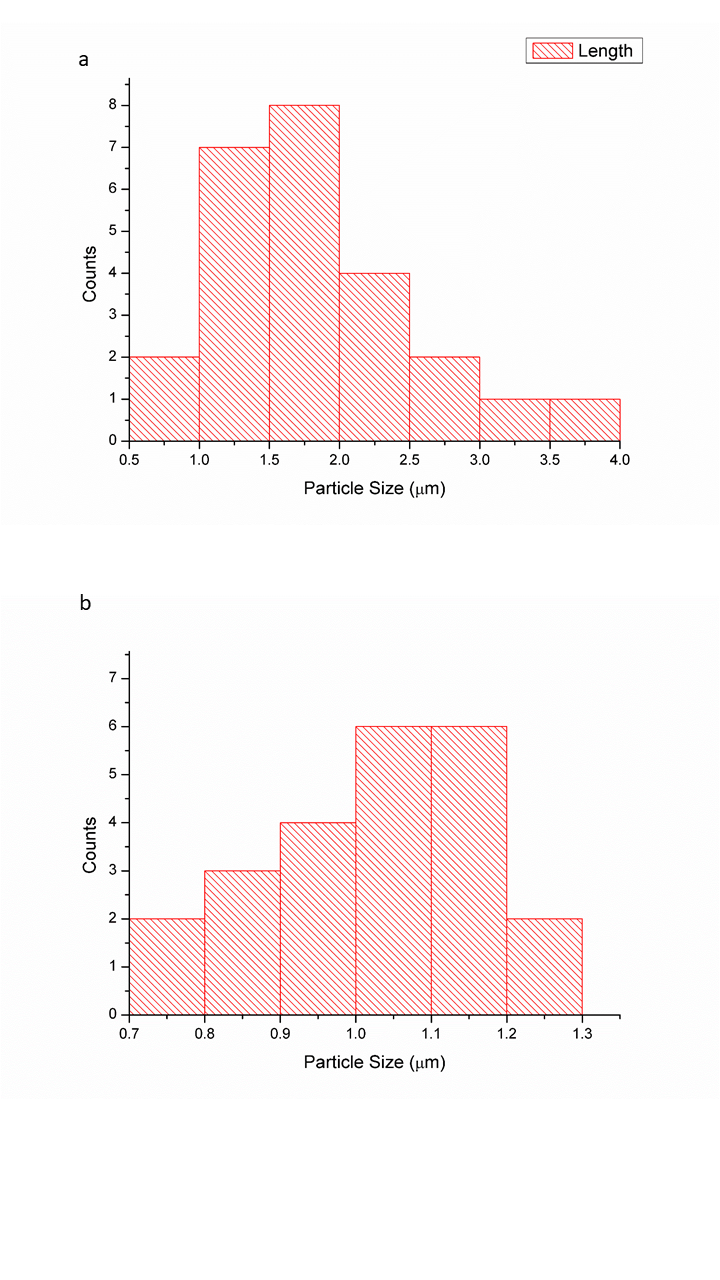
*

**Fig. S1** Average particle size analysis of Fe_2_O_3_ (a) and WO_3_ (b) NPs.
